# Design, Synthesis and Biological Evaluation of Sulfamide and Triazole Benzodiazepines as Novel p53-MDM2 Inhibitors

**DOI:** 10.3390/ijms150915741

**Published:** 2014-09-05

**Authors:** Zhiliang Yu, Chunlin Zhuang, Yuelin Wu, Zizhao Guo, Jin Li, Guoqiang Dong, Jianzhong Yao, Chunquan Sheng, Zhenyuan Miao, Wannian Zhang

**Affiliations:** 1 School of Pharmacy, Ningxia Medical University, Yinchuan, Ningxia 750004, China; E-Mail: zlyu1013@163.com; 2 School of Pharmacy, Second Military Medical University, 325 Guohe Road, Shanghai 200433, China; E-Mails: zclnathan@163.com (C.Z.); wuyuelin517@hotmail.com (Y.W.); guozizhao@126.com (Z.G.); 13601689584@163.com (J.L.); dgq-81@163.com (G.D.); yaojz@sh163.net (J.Y.); shengcq@hotmail.com (C.S.)

**Keywords:** p53-MDM2, small molecule inhibitors, sulfamidebenzodiazepine, triazolebenzodiazepine, antitumor activity

## Abstract

A series of sulfamide and triazole benzodiazepines were obtained with the principle of bioisosterism. The p53-murine double minute 2 (MDM2) inhibitory activity and *in vitro* antitumor activity were evaluated. Most of the novel benzodiazepines exhibited moderate protein binding inhibitory activity. Particularly, triazole benzodiazepines showed good inhibitory activity and antitumor potency. Compound **16** had promising antitumor activity against the U-2 OS human osteosarcoma cell line with an *IC*_50_ value of 4.17 μM, which was much better than that of nutlin-3. The molecular docking model also successfully predicted that this class of compounds mimicked the three critical residues of p53 binding to MDM2.

## 1. Introduction

The p53, a tumor suppressor, plays a critical role in DNA repair, differentiation, senescence, apoptosis, and cell-cycle arrest [1,2]. In approximately 50% of all human cancers, the p53 has been deactivated or deleted [3,4]. The p53 retains its wild-type form in the remaining human cancers and the activity is effectively inhibited through direct interaction with the human murine double minute 2 (MDM2) oncoprotein [5,6,7,8]. Thus, targeting p53-MDM2 protein-protein interaction has emerged as a novel strategy for development of anticancer drugs. Blocking this protein-protein interaction, releasing the p53 and recovering its function are the mechanism and new strategy of cancer therapy [9,10].

**Figure 1:**
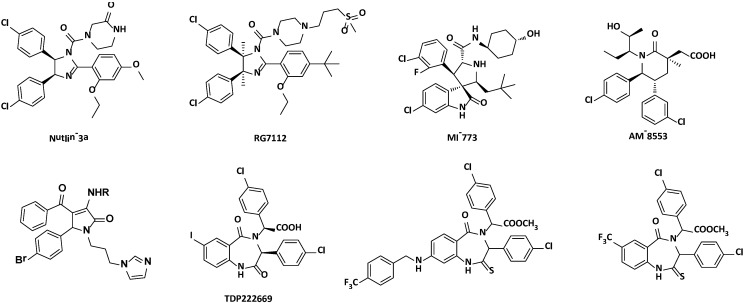
Representative p53-murine double minute 2 (MDM2) inhibitors.

The binding cavities within MDM2, namely Phe19, Trp23, Leu26 pockets, are important hot spots for designing inhibitors that would release p53 [9,11]. Recently, a number of non-peptide small-molecule inhibitors based on the three hot spots have been reported (Figure 1). Inspiringly, six of them have been advanced into Phase I clinical trials [12], such as the nutlins (RG7112) [13,14,15], spiro-oxindoles (MI-773) [16]. Scientists from Amgen have reported another class of potent MDM2 inhibitors featuring a 1,3,5,6-tetrasubstituted piperidinone scaffold, AM-8553 [17]. Very recently, a pyrrolidone scaffold was identified by structure-based design in our group [18]. And a follow-up research was performed to develop a novel pyrrolo[3,4-c]pyrazole scaffold as dual inhibitors of p53-MDM2 interaction and the NF-κB pathway [19]. TDP222669, a benzodiazepine-based inhibitor of p53-MDM2 was reported by Grasberger *et al.* in 2005 [20]. However, this compound suffered from limited bioavailability and rapid *in vivo* clearance [21,22,23]. Thus, a series of thio-benzodiazepines were then designed based on the principle of bioisosterism, possessing both p53-MDM2 inhibitory activity and *in vitro* antitumor activity (Figure 1) [24,25]. 

In this study, we designed a series of sulfamide and triazole benzodiazepines based on the principle of bioisosterism. The triazole benzodiazepines showed good biological activity and could be used as promising lead structures for further optimization.

## 2. Results and Discussion

### 2.1. Chemistry

**Scheme 1:**
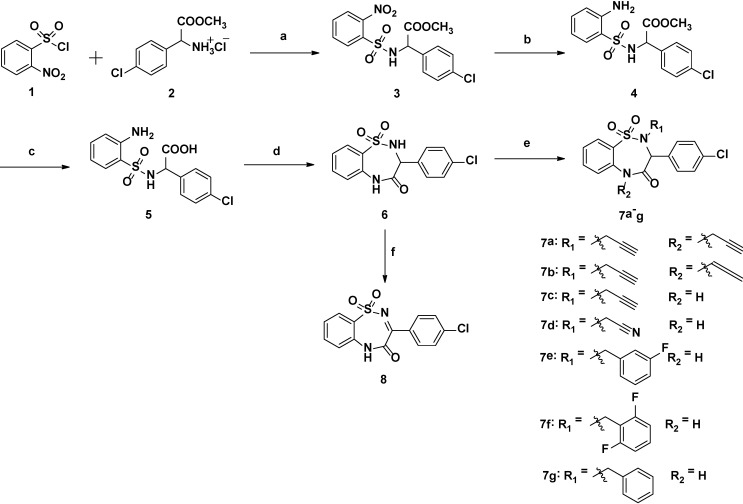
Synthetic route of sulfamide-benzodiazepines.

In this study, *O*-nitrobenzenesulfonyl chloride (**1**) and methyl amino(4-chlorophenyl)acetate hydrochloride (**2**) were used to perform the condensation reaction to get intermediate **3**. Then, the intermediate **6** was made by reduction, hydrolysis and intramolecular condensation. The target compounds (**7a**–**7g**) were obtained by introducing aromatic and aliphatic groups on compound **6** using well-established conditions for substitution reaction. The compound **8** could be obtained without adding haloalkane using the similar condition (see Scheme 1).

Reagents and conditions: (a) *N*,*N*-diisopropylethylamine, DCM, 0–25 °C, overnight; (b) Fe/HOAc, 70 °C, 2.5 h; (c) LiOH, THF:MeOH:H_2_O = 4:2:1, overnight; (d) EDC·HCl, DMAP, overnight; (e) acetonitrile, K_2_CO_3_, RX, 60 °C, 3–6 h; (f) acetonitrile, K_2_CO_3_, 60 °C, 4 h.

**Scheme 2:**
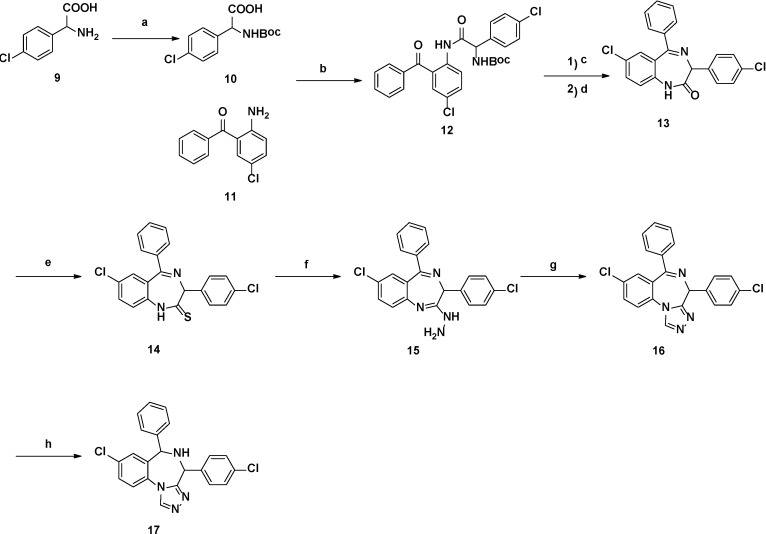
Synthetic route of triazole-benzodiazepines.

For the triazole benzodiazepines, imine **13** was intramolecular cyclized from compound **12** in the weak acid condition, following the HBTU condensation of compounds **10** and **11**. Then, the triazole target compound **16** was synthesized by Lawesson’s reagent, hydrazine hydrate, and cyclization using triethylorthoformate. Compound **16** was then reduced by NaBH_3_CN to get compound **17** (see Scheme 2).

Reagents and conditions: (a) (Boc)_2_O, 1 M NaOH, dioxane, r.t., overnight; (b) HBTU, DMAP, r.t., overnight; (c) TFA, DCM, r.t., overnight; (d) HOAc, DCM, reflux, 5 h; (e) toluene, Lawesson’s Reagent, 70 °C, 4 h; (f) THF, NH_2_NH_2_^.^H_2_O, r.t., overnight; (g) Triethylorthoformate, toluene, reflux, 4 h; (h) NaBH_3_CN, MeOH, HOAc.

### 2.2. Disrupting the Binding of p53-MDM2

**Figure 2:**
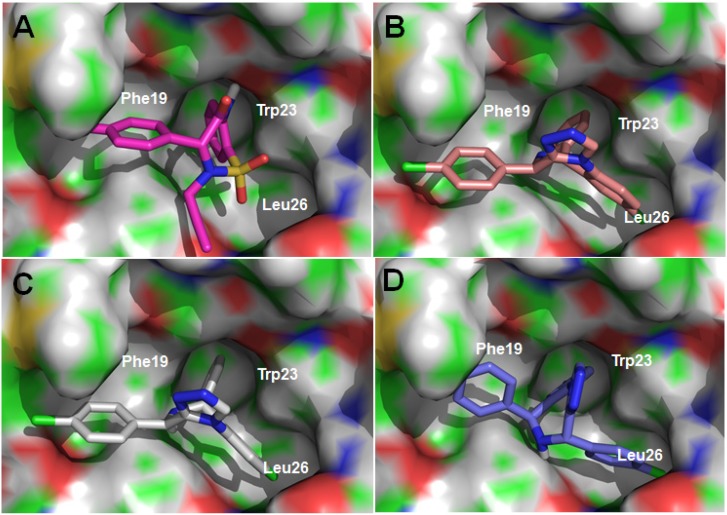
Docking modes of the sulfamide and triazole benzodiazepines with MDM2: (**A**) compound **7c** (R); (**B**) compound **16** (R); (**C**) compound **17** (R, R); (**D**) compound **17** (*S*, *S*).

A well-established fluorescence polarization (FP) binding assay was performed to measure the inhibitory activity (*K*_i_) of designed compounds. Nutlin-3, a commercial available p53-MDM2 inhibitor, was used as the reference compound. As presented in Table 1, most of this series of benzodiazepines exhibited moderate to good inhibitory activity. In order to explore whether the aramatic groups could be substituted by nonaramatic groups, aliphatic substituted compounds **7a**–**7c** were synthesized based on the principle of scaffold hopping, which showed the inhibitory activity with *K*_i_ values in nanomolar range. Particularly, compound **7c** had a *K*_i_ value of 0.26 μM, comparable to the positive compound nutlin-3.The further insight of compound **7c** with MDM2 protein was re-examined by molecular modeling (Figure 2A). The two phenyl rings of (R)-**7c** were located in the Phe19 and Trp23 pockets respectively and the propargyl group was partly inserted into the Leu26 pocket. However, the disubstituted analogues (**7a** and **7b**) were less active, probably due to limited space for binding. For similar reason, the aromatic substituted compounds (**7e** and **7f**) were totally inactive. Only compound with unsubstituted benzyl group (**7g**) showed moderate p53-MDM2 inhibitory activity (*K*_i_ = 8.14 μM). Compound **8**, reduced from compound **6**, was observed dramatic decrease of the inhibitory activity, highlighting the importance of the conformational restriction.

**Table 1:** *In vitro* activity of the designed compounds.

Compounds	*K*_i_ (μM )	*IC*_50_ (μM)			
		Saos-2	U-2 OS	A549	NCI-H1299
		(p53 null)	(wt-p53)	(wt-p53)	(p53 null)
**7a**	0.43	>100	>100	>100	>100
**7b**	0.36	>100	>100	>100	>100
**7c**	0.26	>100	>100	>100	>100
**7d**	>100	>100	>100	92.3	>100
**7e**	>100	>100	90.5	>100	>100
**7f**	78.0	>100	>100	>100	>100
**7g**	8.14	>100	>100	>100	>100
**8**	11.9	>100	>100	>100	>100
**13**	0.20	3.01	3.12	9.16	6.11
**14**	8.76	3.67	5.31	24.05	15.56
**16**	1.22	3.55	4.17	7.62	5.50
**17**	2.80	24.81	33.98	26.78	34.59
**Nutlin-3**	0.09	20.8	16.3	12.7	4.15

For the triazole derivatives, the target compound and intermediates were tested. Unsurprisingly, the benzodiazepine (**13**) showed good activity (*K*_i_ = 0.20 μM) on disrupting the p53-MDM2. The target triazole derivative (**16**) had a moderate *K*_i_ value of 1.22 μM, which is much better than compound **14** and **17**. As shown in Figure 2, the R configuration of compound **16** was predicted to bind to MDM2 protein probably, which was similar to the compound **7c**. Two 4-cholrophenyl groups and the benzene ring, mimicking the three key hydrophobic residues of p53, were inserted into Phe19, Leu26 and Trp23 pockets (Figure 2B). However, only two isomers of target compound **17** could bind well to MDM2 (Figure 2C,D), presumably the reason for the decrease of the activity compared to compound **16**. Interestingly, these two isomers had different binding poses. The (*R*, *R*)-isomer could be inserted into the binding pockets as well as R-compound **16**. For the (*S*, *S*)-isomer, the two 4-chlorophenyl groups were occupied the Trp23 and Leu26 pockets, while the benzene ring was located in Phe19 pocket.

### 2.3. In Vitro Antitumor Activity

To investigate the *in vitro* antiproliferative activity of the designed p53-MDM2 inhibitors, four human tumor cell lines, namely U-2 OS (wild-type p53), A549 (wild-type p53), Saos-2 (p53 null), and NCI-H1299 (p53 null), were chosen for assaying. Nutlin-3 was used as a reference compound. The obtained *IC*_50_ values for the p53-MDM2 inhibitors are summarized in Table 1. Unfortunately, all the sulfamide benzodiazepines showed poor *in vitro* antitumor activity, potentially due to their poor aqueous solubility. Comparing with the totally inactive sulfamide benzodiazepines, the triazole benzodiazepines showed promising antiproliferative activity. Notably, compound **16** showed better activity (*IC*_50_ = 7.62 μM) against the A549 cell line than thio-benzodiazepine **14** (*IC*_50_ = 24.05 μM) and nutlin-3 (*IC*_50_ = 12.7 μM). And its antiproliferative activity (*IC*_50_ = 4.17 μM) against the U-2 OS cell line was about 4-fold better than nutlin-3. Moreover, compound **17** exhibited poor activity against these four cell lines, which matched with the protein binding assay. Nevertheless, these compounds had poor selectivity over cancer cell lines with deleted p53, which inspired us to carry out further optimization to find more active analogues. Focused medicinal chemistry work is in progress to expand the SAR of this series of compounds.

## 3. Experimental Section

### 3.1. Chemistry

All reagents and solvents were purchased from commercial suppliers and used as received unless otherwise stated. ^1^H-NMR and ^13^C-NMR spectra were recorded on a BRUKER AVANCE 300 and 600 spectrometer (Bruker Company, Rheinstetten, Germany), using TMS as an internal standard and CDCl_3_ or DMSO-*d*_6_ as solvents. Chemical shifts (δ values) and coupling constants (*J* values) are given in ppm and Hz, respectively. TLC analysis was carried out on silica gel plates GF254 (QingdaoHaiyang Chemical, Qingdao, China). Flash column chromatography was carried out on silica gel 300–400 mesh. Anhydrous solvent and reagents were all analytical pure and dried through routine protocols.

Methyl 2-(4-chlorophenyl)-2-(2-nitrophenylsulfonamido)acetate (**3**). Methyl 2-amino-2-(4-chlorophenyl)acetate hydrochloride (**2**, 2.15 g, 9.1 mmol) and *N*,*N*-diisopropylethylamine (1.76 g, 13.6 mmol) was added into dry CH_2_Cl_2_ (80 mL). The solution was stirred at 0 °C then *O*-nitrobenzenesulfonyl chloride (**1**, 1.0 g, 4.5 mmol) was added slowly. After that, the mixture was stirred overnight at room temperature and concentrated under vacuum. The residue was purified by flash column chromatography to give compound **3**, yield: 81%. ^1^H-NMR (DMSO-*d*_6_, 600 MHz): δ 9.39 (d, 1H, *J* = 4.3 Hz), 7.95–7.91 (m, 1H), 7.90–7.89 (m, 1H), 7.81–7.79 (m, 1H), 7.77–7.76 (m, 1H), 7.38–7.34 (m, 4H), 5.23 (d, 1H, *J* = 4.3 Hz), 3.54 (s, 3H); ESI-MS (*m*/*z*): 385.52 (M + H)^+^.

Methyl 2-(2-aminophenylsulfonamido)-2-(4-chlorophenyl)acetate (**4**). Compound **3** (1.0 g, 2.6 mmol) was stirred with AcOH (50 mL). The resulting solution was heated at 45 °C and treated under vigorous stirring with iron powder (2.0 g, 35.7 mmol) in one portion. When the exothermic reaction had subsided, the reaction mixture was heated at 65–70 °C for 2.5 h and then allowed to cool and stirred with CH_2_Cl_2_ (50 mL) and H_2_O (50 mL). The resulting suspension was filtered to remove the unreacted iron and the filtrate transferred to a separating funnel. The organic layer was washed with H_2_O (50 mL), NaHCO_3_ (aq, 2%; 50 mL), H_2_O (50 mL) and then separated, dried (Na_2_SO_4_), and evaporated to dryness to give product **4**, 0.88 g, yield: 95%. ^1^H-NMR (DMSO-*d*_6_, 600 MHz): δ 8.79 (d, 1H, *J* = 4.1 Hz), 7.43–7.42 (m, 1H), 7.33–7.32 (m, 2H), 7.29–7.27 (m, 2H), 7.20–7.17 (m, 1H), 6.69–6.53 (m, 1H), 6.53–6.50 (m, 1H), 5.90 (s, 2H), 4.96 (d, 1H, *J* = 4.1 Hz), 3.48 (s, 3H); ESI-MS (*m*/*z*): 355.67 (M + H)^+^.

3-(4-Chlorophenyl)-2,3-dihydrobenzo[*f*][1,2,5]thiadiazepin-4(5*H*)-one 1,1-dioxide (**6**). Compound **4** (0.50 g, 1.4 mmol) was dissolved in a mixed solution (THF:MeOH:H_2_O = 4:2:1, 35 mL). Then, LiOH (0.10 g, 4.2 mmol) was added and the mixture was stirred overnight at room temperature. The resulting solution was evaporated to dryness and dissolved in H_2_O (30 mL), extracted by ether (20 mL × 2). The aqueous layer was acidified with HCl (1 mol/L) to reach pH 4. The resulting solid was filtered and then directly dissolved in CH_2_Cl_2_ (40 mL) with EDC·HCl (0.54 g, 2.8 mmol) and DMAP (0.05 g, 0.4 mmol), stirred overnight at room temperature and concentrated under vacuum. The residue was purified by flash column chromatography to give compound **6**, yield: 71%. ^1^H-NMR (DMSO-*d*_6_, 600 MHz): δ 10.49 (s, 1H), 9.14 (d, 1H, *J* = 8.4 Hz), 7.81–7.79 (m, 1H), 7.63–7.60 (m, 1H), 7.43–7.42 (m, 2H), 7.39–7.37 (m, 2H), 7.31–7.29 (m, 2H), 5.29 (d, 1H, *J* = 8.0 Hz); ESI-MS (*m*/*z*): 323.58 (M + H)^+^.

### 3.2. General Procedure

#### 3.2.1. Synthesis of **7a** and **7b**

Compound **6** (0.20 g, 0.6 mmol), haloalkane (0.35 g, 2.9 mmol) and potassium carbonate (0.09 g, 0.6 mmol) were mixed in acetonitrile (10 mL) and stirred at 60 °C for 5 h. The resulting precipitate was filtered off and the filtrate was then evaporated to dryness. The residue was purified by flash column chromatography to give two compounds. The compound with lower polarity was **7a** (yield: 25%); compound **7b** was the tautomer of compound **7a** (yield: 25%).

#### 3.2.2. Synthesis of **7c**–**7g**

Compound **6** (0.20 g, 0.6 mmol), haloalkane (0.07 g, 0.58 mmol) and potassium carbonate (0.09 g, 0.6 mmol) were mixed in acetonitrile (10 mL) and stirred at 60 °C for 5 h. The resulting precipitate was filtered off and the filtrate was then evaporated to dryness. The residue was purified by flash column chromatography to give compounds **7c**–**7g**, yield: 25%–49%.

3-(4-Chlorophenyl)-2,5-di(prop-2-yn-1-yl)-2,3-dihydrobenzo[*f*][1,2,5]thiadiazepin-4(5*H*)-one 1,1-dioxide (**7a**). ^1^H-NMR (DMSO-*d*_6_, 600 MHz): δ 7.83 (dd, 1H, *J*_1_ = 1.44 Hz, *J*_2_ = 8.01 Hz), 7.49 (t, 1H, *J* = 7.65 Hz), 7.35 (t, 1H, *J* = 7.59 Hz), 7.15–7.09 (m, 3H), 6.89 (s, 2H), 5.96 (d, 1H, *J* = 6.10 Hz), 5.84 (s, 1H), 4.77 (dd, 1H, *J* = 2.38, 17.93 Hz), 4.66 (s, 1H), 4.50–4.47 (m, 1H), 4.19 (d, 1H, *J* = 14.05 Hz), 3.29 (s, 1H). ^13^C-NMR (150 MHz, DMSO-*d*_6_): δ 168.13, 138.78, 137.67, 135.04, 132.25, 131.89, 130.53, 128.09, 126.93, 126.52, 125.51, 124.27, 124.04. 78.91, 78.34, 75.31; ESI-MS (*m*/*z*): 399.82 (M + H)^+^.

3-(4-Chlorophenyl)-2-(prop-2-yn-1-yl)-5-(propa-1,2-dien-1-yl)-2,3-dihydrobenzo[*f*][1,2,5]thiadiazepin-4(5*H*)-one 1,1-dioxide (**7b**). ^1^H-NMR (DMSO-*d*_6_, 600 MHz): δ 7.91–7.86 (m, 2H), 7.81 (s, 1H), 7.59 (t, 1H, *J* = 7.86 Hz), 7.44 (d, 2H, *J* = 7.98 Hz), 7.34 (s, 2H), 4.65–4.56 (m, 3H), 4.18 (d, 1H, *J* = 18.93 Hz), 3.24 (d, 1H, *J* = 18.26 Hz), 3.13 (t, 1H, *J* = 2.44 Hz), 3.01 (s, 1H); ^13^C-NMR (75 MHz, DMSO-*d*_6_): δ 165.68, 138.94, 135.68, 134.04, 133.36, 131.76, 130.23, 128.86, 128.23, 128.05, 125.84,78.98, 78.80,75.66; ESI-MS (*m*/*z*): 399.82 (M + H)^+^.

3-(4-Chlorophenyl)-2-(prop-2-yn-1-yl)-2,3-dihydrobenzo[*f*][1,2,5]thiadiazepin-4(5*H*)-one 1,1-dioxide (**7c**). ^1^H-NMR (DMSO-*d*_6_, 600 MHz): δ 10.62 (s, 1H), 7.79–7.77 (m, 1H), 7.71–7.68 (m, 1H), 7.43 (d, 2H, *J* = 8.12 Hz), 7.37–7.33 (m, 4H), 7.24 (d, 1H, *J* = 8.12 Hz), 4.94 (s, 1H), 4.17 (dd, 1H, *J* = 2.3, 18.54 Hz), 3.40 (dd, 1H, *J* = 2.3, 18.54 Hz); ^13^C-NMR (75 MHz, DMSO-*d*_6_): δ 167.89, 136.38, 135.18, 134.80, 133.61, 131.44, 129.72, 128.73, 127.75, 125.09, 123.52, 78.46, 75.35, 64.14; ESI-MS (*m*/*z*): 361.55 (M + H)^+^.

2-(3-(4-Chlorophenyl)-1,1-dioxido-4-oxo-4,5-dihydrobenzo[*f*][1,2,5]thiadiazepin-2(3*H*)-yl)acetonitrile (**7d**). ^1^H-NMR (DMSO-*d*_6_, 600 MHz): δ 10.75 (s, 1H), 7.82 (dd, 1H, *J*_1_ = 1.57 Hz, *J*_2_ = 8.04 Hz), 7.75–7.72 (m, 1H), 7.45 (d, 2H, *J* = 8.47 Hz), 7.41–7.38 (m, 3H), 7.28 (d, 1H, *J* = 8.07 Hz), 5.06 (s, 1H), 4.34 (d, 1H, *J* = 18.58 Hz), 3.96 (d, 1H, *J* = 18.58 Hz); ESI-MS (*m*/*z*): 362.29 (M + H)^+^.

3-(4-Chlorophenyl)-2-(3-fluorobenzyl)-2,3-dihydrobenzo[*f*][1,2,5]thiadiazepin-4(5*H*)-one 1,1-dioxide (**7e**). ^1^H-NMR (DMSO-*d*_6_, 300 MHz): δ 10.63 (s, 1H), 7.79–7.76 (m, 1H), 7.68–7.62 (m, 1H), 7.32–7.17 (m, 7H), 6.97–6.76 (m, 3H), 5.28 (s, 1H), 4.24 (s, 2H); ^13^C-NMR (75 MHz, DMSO-*d*_6_): δ 169.06, 138.90, 138.80, 136.23, 135.99, 134.87, 132.99, 131.24, 130.55, 129.99, 128.25, 127.13, 124.73, 124.40, 122.92, 115.48, 115.19, 114.81, 114.54, 67.35, 53.29; ESI-MS (*m*/*z*): 431.66 (M + H)^+^.

3-(4-Chlorophenyl)-2-(2,6-difluorobenzyl)-2,3-dihydrobenzo[*f*][1,2,5]thiadiazepin-4(5*H*)-one 1,1-dioxide (**7f**). ^1^H-NMR (DMSO-*d*_6_, 300 MHz): δ 10.56 (s, 1H), 7.86–7.61 (m, 3H), 7.56–7.34 (m, 7H), 6.85–6.82 (m, 1H), 5.11 (s, 1H), 4.40 (d, 1H, *J* = 14.4 Hz), 4.24 (d, 1H, *J* = 14.4 Hz); ESI-MS (*m*/*z*): 449.82 (M + H)^+^.

2-Benzyl-3-(4-chlorophenyl)-2,3-dihydrobenzo[*f*][1,2,5]thiadiazepin-4(5*H*)-one 1,1-dioxide (**7g**). ^1^H-NMR (DMSO-*d*_6_, 600 MHz): δ 10.55 (s, 1H), 7.77 (dd, 1H, *J*_1_ = 1.41 Hz, *J*_2_ = 7.79 Hz,) 7.63–7.60 (m, 1H), 7.29–7.26 (m, 3H), 7.18–7.17 (m, 3H), 7.14–7.12 (m, 3H), 7.02–7.00 (m, 2H), 5.17 (s, 1H), 4.28 (d, 1H, *J* = 15.44 Hz), 4.16 (d, 1H, *J* = 15.44 Hz); ^13^C-NMR (75 MHz, DMSO-*d*_6_): δ 169.14, 135.96, 135.58, 131.09, 130.26, 128.84, 128.56, 128.29, 127.99, 127.04, 124.38, 122.83, 67.08, 53.64; ESI-MS (*m*/*z*): 413.61 (M + H)^+^.

3-(4-Chlorophenyl)benzo[*f*][1,2,5]thiadiazepin-4(5*H*)-one 1,1-dioxide (**8**). Compound **6** (0.10 g, 0.3 mmol), and potassium carbonate (0.05 g, 0.36 mmol) were mixed in acetonitrile (10 mL) and stirred at 60 °C for 4 h. The resulting precipitate was filtered off and the filtrate was then evaporated to dryness. The residue was purified by flash column chromatography to give compound **8**, yield: 50%. ^1^H-NMR (DMSO-*d*_6_, 600 MHz): δ 8.14–8.12 (m, 2H), 7.88 (dd, 1H, *J*_1_ = 1.19 Hz, *J*_2_ = 8.12 Hz), 7.76–7.73 (m, 1H), 7.70–7.67 (m, 3H), 7.55–7.52 (m, 1H), 7.70–7.67 (m, 1H); ^13^C-NMR (75 MHz, DMSO-*d*_6_): δ 149.80, 140.28, 135.16, 134.06, 133.23, 132.09, 129.31, 127.91,124.01,119.37; ESI-MS (*m*/*z*): 321.33 (M + H)^+^.

*tert*-Butyl (2-((2-benzoyl-4-chlorophenyl)amino)-1-(4-chlorophenyl)-2-oxoethyl)carbamate (**12**). 4-chlorophenylglycine (**9**, 5.00 g, 48.6 mmol) was dissolved in 1,4-dioxane (60 mL) and NaOH (1 mol/L, 30 mL). Di-*tert*-butyl dicarbonate (9.2 g, 42.2 mmol) was then added into the mixture and stirred at room temperature for 12 h. After completion of reaction, the solution mixture was then acidified with HCl (1 mol/L) to reach pH 5, extracted by CH_2_Cl_2_ (100 mL × 3). The organic layer was then evaporated to dryness to get crude product 10 which was directly used in the next step. Compound **10**, HBTU (11.2 g, 29.5 mmol), DMAP (0.66 g, 5.4 mmol) and compound 11 (6.24 g, 27.0 mmol) was dissolved in CH_2_Cl_2_ (200 mL) and stirred at room temperature for 12 h. The resulting mixture was evaporated to dryness. The residue was purified by flash column chromatography to give compound **12**, 2.8 g yield: 21%. ^1^H-NMR (CDCl_3_, 300 MHz): δ 11.17 (s, 1H), 8.58 (d, 1H, *J* = 9.6 Hz), 7.67–7.61 (m, 3H), 7.53–7.51 (m, 3H), 7.41 (d, 2H, *J* = 8.2 Hz), 7.34 (d, 2H, *J* = 8.2 Hz), 7.26 (s, 1H), 5.76 (s, 1H), 5.27 (s, 1H), 1.4 (s, 9H); ESI-MS (*m*/*z*): 500.31 (M + H)^+^.

7-Chloro-3-(4-chlorophenyl)-5-phenyl-1*H*-benzo[*e*][1,4]diazepin-2(3*H*)-one (**13**). Compound **12** (0.4 g, 0.8 mmol) was added into CH_2_Cl_2_ (15 mL) with TFA (0.37 g, 3.2 mmol) and stirred overnight at room temperature. The mixture was then concentrated to dry and dissolved in CH_2_Cl_2_ (10 mL). Five drops of TFA was then added as the catalyst. The mixture was heated to reflux for 2 h. The resulting mixture was evaporated to dryness. The residue was purified by flash column chromatography to give compound **13**, 0.25 g, yield: 83%. ^1^H-NMR (CDCl_3_, 600 MHz): δ 10.86 (s, 1H), 7.69 (dd, 1H, *J*_1_ = 2.46 Hz, *J*_2_ = 8.76 Hz), 7.60 (d, 2H, *J* = 8.4 Hz), 7.53–7.51 (m, 3H), 7.47–7.43 (m, 4H), 7.34 (d, 1H, *J* = 8.76 Hz), 7.30 (d, 1H, *J* = 2.46 Hz), 4.87 (s, 1H); ^13^C-NMR (75 MHz, DMSO-*d*_6_): δ 169.94, 167.32, 138.88, 138.84, 138.13, 132.43, 132.26, 131.47, 131.08, 130.05, 129.86, 128.94, 128.20, 127.16, 123.84, 67.23; ESI-MS (*m*/*z*): 381.05 (M + H)^+^.

7-Chloro-3-(4-chlorophenyl)-5-phenyl-1*H*-benzo[*e*][1,4]diazepine-2(3*H*)-thione (**14**). A dry toluene solution of compound **13** (0.20 g, 0.53 mmol) under an atmosphere of nitrogen was treated with Lawesson’s Reagent (2,4-*bis*(4-methoxyphenyl)-2,4-disulfide, 0.22 g, 0.54 mmol) under 70 °C for 3 h, cooled to room temperature, concentrated under vacuum and purified by column chromatography to give **14**, 0.16 g, yield: 77%. ^1^H-NMR (CDCl_3_, 600 MHz): δ 12.67 (s, 1H), 7.74 (dd, 1H, *J*_1_ = 2.46 Hz, *J*_2_ = 8.82 Hz), 7.60 (d, 2H, *J* = 8.4 Hz), 7.55–7.52 (m, 3H), 7.49–7.46 (m, 3H), 7.41 (d, 2H, *J* = 8.4 Hz), 7.34 (d, 1H, *J* = 2.4 Hz), 5.15 (s, 1H); ^13^C-NMR (75 MHz, DMSO-*d*_6_): δ 201.57, 166.77,140.04, 139.25, 138.42, 132.36, 131.86, 131.28, 130.24, 129.95, 129.50, 129.00, 128.80, 127.88, 124.24, 71.22, 26.80; ESI-MS (*m*/*z*): 397.28 (M + H)^+^.

8-Chloro-4-(4-chlorophenyl)-6-phenyl-4*H*-benzo[*f*][1,2,4] (**16**). Compound **14** was added into THF (15 mL). The resulting solution was then treated with 85% hydrazine hydrate at 0 °C for 1 h, and then stirred at room temperature for 12 h. After completion of reaction, the mixture was evaporated to dryness to afford the crude product 15 which was directly added into a dry toluene (15 mL) solution of triethylorthoformate (10 mL). The mixture was then stirred under reflux for 2 h. The resulting mixture was then evaporated to dryness. The residue was purified by flash column chromatography to give compound **16**, 0.14 g yield: 71%. ^1^H-NMR (CDCl_3_, 600 MHz): δ 9.20 (s, 1H), 7.99–7.95 (m, 2H), 7.77 (d, 2H, *J* = 8.46 Hz), 7.52–7.49 (m, 5H), 7.47 (d, 1H, *J* = 2.16 Hz), 7.45–7.43 (m, 2H), 5.61 (s, 1H); ^13^C-NMR (75 MHz, DMSO-*d*_6_): δ 166.26, 156.03, 143.31, 139.22, 137.37, 133.07, 132.86, 132.82, 131.81, 131.23, 129.87, 128.96, 128.86, 128.50, 125.80, 58.74; ESI-MS (*m*/*z*): 405.32 (M + H)^+^.

8-Chloro-4-(4-chlorophenyl)-6-phenyl-5,6-dihydro-4*H*-benzo[*f*][1,2,4]triazolo[4,3-a][1,4]diazepine (**17**). Glacial acetic acid (3 mL) and **16** (0.05 g, 0.12 mmol) were mixed in CH_3_OH (10 mL). Then, NaBH_3_CN (0.03 g, 0.48 mmol) was then added to the solvent, stirred for 12 h. Removal of the solvent under reduced pressure and the residue was purified by column chromatography to give **17**, 0.04 g, yield: 80%. ^1^H-NMR (CDCl_3_, 600 MHz): δ 8.87 (s, 1H), 7.63 (d, 1H, *J* = 8.46 Hz), 7.56 (dd, 1H, *J*_1_ = 2.34 Hz, *J*_2_ = 8.46 Hz), 7.43 (d, 2H, *J* = 8.52 Hz), 7.33–7.29 (m, 7H), 7.01 (d, 1H, *J* = 2.42 Hz), 5.29 (d, 1H, *J* = 7.81 Hz), 5.19 (d, 1H, *J* = 7.81 Hz), 4.32 (t, 1H, *J* = 7.52 Hz); ^13^C-NMR (75 MHz, DMSO-*d*_6_): δ 153.29, 143.09, 141.91, 139.73, 138.26, 133.03, 132.49, 132.20, 130.53, 130.45, 129.85, 129.21, 128.58, 128.25, 127.34, 125.24, 59.74, 53.31; ESI-MS (*m*/*z*): 407.51 (M + H)^+^.

### 3.3. Computational Protocol

Molecular docking was used to predict the binding mode of the synthesized benzodiazepine derivatives. The crystal structure of MDM2 (PDB code: 1T4E) was prepared by removing the benzodiazepine and adding hydrogen atoms in GOLD 5.0. We used TDP222669 as a positive control. The active site was defined to encompass all MDM2 atoms within a 12 Å radius sphere from the center of 1T4E ligand. Other parameters were set by default.

### 3.4. p53-MDM2 Binding Assay

The dose-dependent binding experiments were carried out with serial dilution in DMSO of compounds. A 5 µL sample of the tested sample and preincubated (for 30 min) MDM2 binding domain (1–118) (10 nM) and PMDM6-F peptide (Anaspec, Fremont, CA, USA) (10 nM) in the assay buffer (100 mM potassium phosphate, pH 7.5; 100 µg/mL bovine gamma globulin; 0.02% sodium azide) were added into black 96-well microplates with F-bottom and chimney wells (Corning #3993) to produce a final volume of 115 µL. For each assay, the controls included the MDM2 binding domain and PMDM6-F. The polarization values were measured after 1 h of incubation at room temperature using Biotek Synergy H2 with a 480 nm excitation filter, a 528 nm static and polarized filter (BioTek, Winooski, VT, USA). The *K*_i_ values were determined from a plot using nonlinear least-squares analysis. And curve fitting was performed using GraphPad Prism software. Nutlin-3 (Sigma-Aldrich, Shanghai, China) was used as reference compound for validating the assay in each plate [18,19,24,25,26,27].

### 3.5. In Vitro Antitumor Activity

The cellular growth inhibitory activity was determined using four human cancer cell lines [U-2 OS (wild-type p53), A549 (wild-type p53), Saos-2 (p53 null), and NCI-H1299 (p53 null)]. 5–6 × 10^4^ cells per well were plated in 96-well plates. After culturing for 24 h, test compounds were added onto triplicate wells with different concentrations and 0.1% DMSO for control. After 72 h of incubation, 20 μL of MTT (3-[4,5-dimethylthiazol-2-yl]-2,5-diphenyltetrazoliumbromide) solution (5 mg/mL) was added to each well, and after the samples were shaken for 1min the plate was incubated further for 4 h at 37 °C. Benzodiazepines were dissolved with 100 μL of DMSO. The absorbance (OD) was quantitated with microplate using Biotek Synergy H2 at 570 nm (BioTek, Winooski, VT, USA). Wells containing no drugs were used as blanks. Concentration of the compounds that inhibited cell growth by 50% (*IC*_50_) was calculated [28].

## 4. Conclusions

A series of novel sulfamide and triazole benzodiazepines inhibitors of p53-MDM2 protein-protein interaction were successfully obtained by the principle of bioisosterism. Most of the novel benzodiazepines exhibited moderate protein binding inhibitory activity. Particularly, triazole benzodiazepines showed good inhibitory activity and antitumor potency. The molecular docking model also successfully predicted that this class of compounds mimicked the three critical residues of p53 binding to MDM2. In conclusion, this class of benzodiazepines can be used as promising lead structures for further optimization.
